# Bifunctional Cellulose Interlayer Enabled Efficient Perovskite Solar Cells with Simultaneously Enhanced Efficiency and Stability

**DOI:** 10.1002/advs.202207202

**Published:** 2023-02-07

**Authors:** Zilong Zhang, Can Wang, Feng Li, Lusheng Liang, Liulian Huang, Lihui Chen, Yonghao Ni, Peng Gao, Hui Wu

**Affiliations:** ^1^ College of Material Engineering, National Forestry and Grassland Administration Key Laboratory of Plant Fiber Functional Materials Fujian Agriculture and Forestry University Fuzhou Fujian 350108 P. R. China; ^2^ State Key Laboratory of Structural Chemistry Fujian Institute of Research on the Structure of Matter Chinese Academy of Sciences 350002 Fuzhou P. R. China; ^3^ Xiamen Key Laboratory of Rare Earth Photoelectric Functional Materials Xiamen Institute of Rare Earth Materials Chinese Academy of Sciences 361021 Xiamen P. R. China; ^4^ Laboratory for Advanced Functional Materials Xiamen Institute of Rare Earth Materials Chinese Academy of Sciences 361021 Xiamen P. R. China; ^5^ University of Chinese Academy of Sciences 100049 Beijing P. R. China; ^6^ Limerick Pulp and Paper Centre, Department of Chemical Engineering University of New Brunswick NBE3B 5A3 Fredericton Canada

**Keywords:** bifunctional cellulose derivative, charge transport, defect passivation, interfacial layer, perovskite solar cell, stability

## Abstract

Interfacial engineering is a vital strategy to enable high‐performance perovskite solar cells (PSCs). To develop efficient, low‐cost, and green biomass interfacial materials, here, a bifunctional cellulose derivative is presented, 6‐O‐[4‐(9H‐carbazol‐9‐yl)butyl]‐2,3‐di‐O‐methyl cellulose (C‐Cz), with numerous methoxy groups on the backbone and redox‐active carbazole units as side chains. The bifunctional C‐Cz shows excellent energy level alignment, good thermal stability and strong interactions with the perovskite surface, all of which are critical for not only carrier transportation but also potential defects passivation. Consequently, with C‐Cz as the interfacial modifier, the PSCs achieve a remarkably enhanced power conversion efficiency (PCE) of 23.02%, along with significantly enhanced long‐term stability. These results underscore the advantages of bifunctional cellulose materials as interfacial layers with effective charge transport properties and strong passivation capability for efficient and stable PSCs.

## Graphical abstract

A bifunctional cellulose derivative, 6‐O‐[4‐(9H‐carbazol‐9‐yl)butyl]‐2,3‐di‐O‐methyl cellulose (C‐Cz), with decent carrier transportation and superior passivation effect is developed. When utilized as the interfacial modifier in perovskite solar cells, a remarkably enhanced efficiency of 23.02% is achieved along with significantly improved long‐term stability.

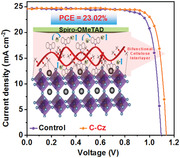

## 1 Introduction

With continuous efforts on the composition engineering of perovskites and device architecture optimization, the power conversion efficiency (PCE) of perovskite solar cells (PSCs) has dramatically increased from the initial 3.8% to a certified value of 25.7%.^[^ ^1^, ^2^, ^3^, ^4^, ^5^, ^6^, ^7^, ^8^, ^9^, ^10^, ^11^ ^]^ Despite the great potential in commercial applications, PSCs still suffer from long‐term stability challenges. Specifically, the solution‐processed polycrystalline perovskite films inevitably generate a high density of defects at the surface and grain boundaries, such as uncoordinated Pb^2+^ and halide ionic defects.^[^ ^12^, ^13^ ^]^ These defects act as charge traps that can aggravate non‐radiative recombination and ion migration, seriously affecting device efficiency and stability.^[^ ^14^, ^15^, ^16^ ^]^ To overcome these issues, numerous strategies, such as additive engineering, compositional modulation and interfacial engineering, have been developed.^[^ ^17^, ^18^, ^19^ ^]^

Generally, organic small molecules and polymers with different functionalities can serve as effective modifiers for optimizing interfacial contact at perovskite grain boundaries or perovskite/hole‐transporting material (HTM) interface.^[^ ^13^, ^14^, ^15^, ^20^, ^21^, ^22^ ^]^ Compared with small molecules, polymers are found to be beneficial for durable defect passivation under a moisture atmosphere owing to their long‐chain molecular structure and abundant functional group sites.^[^ ^15^ ^]^ For example, poly(vinyl acetate),^[^ ^15^ ^]^ poly(ethylene glycol) diacrylate,^[^ ^23^ ^]^ poly(methyl methacrylate),^[^ ^24^ ^]^ poly(4‐vinylpyridine)^[^ ^25^ ^]^ and hetero‐cycle‐based (thiophene,^[^ ^20^ ^]^ quinoxaline,^[^ ^14^ ^]^ benzothiadiazole^[^ ^22^ ^]^ and bithiophene imide^[^ ^26^ ^]^) conjugated polymers, have been reported as adequate interfacial layers for high‐performance PSCs with good stability. However, most polymeric materials need tedious synthetic routes and suffer poor batch‐to‐batch variations, which may result in increased cost and poor device repeatability.

With global climate change and resource shortage in recent years, sustainable and renewable biomass materials have received significant attention.^[^ ^27^, ^28^, ^29^ ^]^ Typically, cellulose (one of the most common natural polymers) and its derivatives containing numerous hydroxyl groups have become vigorously developed materials due to their merits of being non‐toxic, renewable, eco‐friendly, and cost‐effective.^[^ ^30^, ^31^ ^]^ The hydroxyl groups on anhydroglucose units are suited for various chemical modifications, which have shown wide applications in the fields of adhesives, hydrogels, electronic devices, photovoltaic devices, etc.^[^ ^32^, ^33^, ^34^, ^35^, ^36^, ^37^, ^38^, ^39^, ^40^, ^41^ ^]^ Due to the electron lone pairs of the O atoms and the rich hydrogen‐bonding interactions, the cellulose derivatives such as ethyl cellulose,^[^ ^39^ ^]^ cellulose acetate,^[^ ^40^ ^]^ hydroxyalkyl cellulose^[^ ^35^ ^]^ and cellulose acetate butyrate^[^ ^34^ ^]^ have been introduced into the PSCs to passivate the defects at the perovskite grain boundaries and to suppress the non‐radiative recombination, thereby improving device stability. Nevertheless, the intrinsic electrically insulating properties of cellulose derivatives still limit the device performance in PSCs. Therefore, the development of conductive cellulose derivatives as interfacial layers with both charge‐transporting capacity and defect passivation functionality is urgently needed.

Herein, we developed a novel cellulose derivative 6‐*O*‐[4‐(9H‐carbazol‐9‐yl)butyl]‐2,3‐di‐*O*‐methyl cellulose (C‐Cz) as a bifunctional interlayer between the perovskite and hole‐transporting layers. Benefiting from the regioselectively controlled introduction of methyl or carbazole moiety, the resulting C‐Cz possesses two different functional groups in the side chains, which can facilitate simultaneous charge extraction/transport and defects passivation. The C‐Cz shows excellent energy level alignment, proper hole mobility and strong *π*–*π* interactions, guaranteeing efficient hole extraction/transport. Furthermore, the methoxy groups and resultant hydrogen bonds are favorable to passivate defects on the perovskite surface, effectively suppressing the carrier recombination. Surprisingly, the PSCs with C‐Cz as an interlayer achieved a remarkably improved PCE of 23.02%. Significantly, the C‐Cz‐modified PSCs exhibit superior long‐term stability without encapsulation, with 88% of the initial PCE retained after 2800 h in ambient air (≈30% RH). Furthermore, the C‐Cz‐based devices maintain 94% of their initial PCE after storing at 60 °C in N_2_ for 96 h. This work aims to develop new bifunctional low‐cost cellulose‐based interfacial materials to improve the efficiency and stability of PSCs.

## 2 Results and Discussion

**Figure 1:**
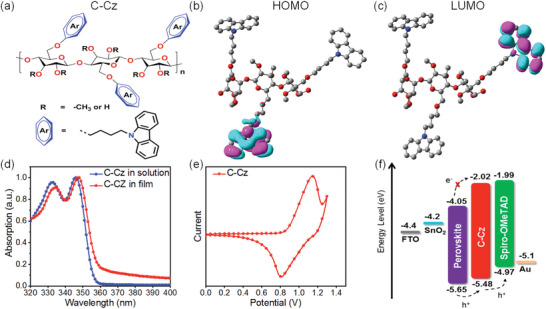
a) Chemical structures, b) the highest occupied molecular orbitals (HOMO), and c) the lowest unoccupied molecular orbitals (LUMO) of C‐Cz. d) Normalized ultraviolet–visible absorption spectra of C‐Cz in chloroform solution and the solid film. e) Cyclic voltammograms of C‐Cz in acetonitrile with the 0.1 m Bu_4_NPF_6_ supporting electrolyte. Scan speed: 100 mV s^−1^, potentials versus Fc/Fc^+^. f) Energy‐level diagram for PSC device.

The chemical structure and synthetic routes of C‐Cz are shown in **Figure** **1**a and Scheme S1, Supporting Information. C‐Cz was synthesized in four steps from extracted cellulose, delivering a yield as high as 73%. 9‐(4‐bromobutyl)‐9H‐carbazole was synthesized according to previous work.^[^ ^42^ ^]^ The introduction of functional groups can be controlled, that is, methyl at *O*‐2 and *O*‐3 positions, and carbazole at the *O*‐6 position, respectively.^[^ ^32^, ^33^ ^]^ All detailed synthetic procedures and methods can be found in the Supporting Information. The chemical structures of C‐Cz were confirmed by the ^1^H NMR and ^13^C nuclear magnetic resonance (NMR) spectra (Figures S1–S3, Supporting Information). The degree of substitution (DS) of the carbazole unit in C‐Cz was almost 1.0, as calculated from the integration values of the peaks corresponding to aromatic and cellulose ring protons in the ^1^H NMR spectra. Furthermore, the introduction of the carbazole group was also identified by the Fourier transform infrared (FTIR) spectra (Figure S4, Supporting Information). The characteristic vibrations of carbazole at 3054, 1613, 1599, 1485, 1452, 1326, 745, and 721 cm^−1^ can be observed clearly. Meanwhile, the weak intensity of the hydroxyl bands at 3420 cm^−1^ in C‐Cz indicates that relatively high degrees of methyl substitution are achieved. The solubility of C‐Cz was measured in common solvents. As shown in Table S1, Supporting Information, C‐Cz exhibited good solubility in common solvents, such as dichloromethane, chloroform, tetrahydrofuran, DMF, DMSO, etc., hence ensuring good solution processability. Notably, starting from very cheap cellulose, the laboratory synthesis cost of C‐Cz is estimated to be ≈33.1 US$ g^−1^ (Table S2, Supporting Information), which is much cheaper than those widely used synthetic polymers bearing hole transport ability (e.g., PTAA, 423 US$ g^−1^; P3HT, 500 US$ g^−1^).^[^ ^43^, ^44^ ^]^ Thermal gravimetric analysis (TGA) and differential scanning calorimetry (DSC) were performed to explore the thermal stability of C‐Cz. C‐Cz shows a thermal decomposition temperature (*T*_d_) over 310 °C (Figure S5a, Supporting Information), indicating good thermal stability. The glass transition temperatures (*T*_g_) was estimated to be 71 °C for C‐Cz (Figure S5b, Supporting Information), which is much higher than that of doped 2,2',7,7'‐tetrakis(N,N‐di‐p‐methoxyphenylamine)‐9,9'‐spirobifluorene (spiro‐OMeTAD) (≈50 °C),^[^ ^45^ ^]^ further confirming the relatively higher thermal stability.

The frontier molecular orbitals of C‐Cz were investigated by density functional theory (DFT) calculations at the level of B3LYP/6‐31G(d,p). For the sake of simplicity, three repeating units were included in the calculation. As shown in Figure 1b,c, the highest occupied molecular orbitals (HOMO) and the lowest unoccupied molecular orbitals (LUMO) are localized on different carbazole units, respectively, which indicates that charge migration mechanism within the C‐Cz can be attributed to the hole hopping between neighboring carbazole units along on the cellulose skeleton.

The normalized ultraviolet–visible (UV–vis) absorption spectra of C‐Cz as solutions in chloroform solution and thin film are shown in Figure 1d, and the corresponding data are summarized in **Table** **1**. C‐Cz exhibit two distinct absorption peaks in chloroform solution and thin film. Compared to the absorption in solution, the absorption maxima of C‐Cz as the thin film shows a redshift of 2 at 348 nm. This might be ascribed to *π*–*π* stacking of the carbazole units.^[^ ^46^ ^]^ The optical energy band gap (*E*_g_^opt^) of C‐Cz was calculated to be 3.46 eV from the absorption onset wavelength.

**Table 1:** Decomposition temperatures, optical properties, frontier energy levels, and hole mobilities of C‐Cz

	*T*_d_ [°C]	*λ*_max_^sol^ [nm]^a)^	*λ*_max_^film^ [nm]^b)^	*λ*_onset_ [nm]^c)^	*E_g_* ^opt^ [eV]^d)^	*E*_HOMO_ [eV]^e)^	*E*_LUMO_ [eV]^f)^	µ_h_ [cm^2^ V^−1^·s^−1^]^g)^
C‐Cz	311	346	348	358	3.46	−5.48	−2.02	1.45 × 10^−5^

^a)^ 5 × 10^−5^ m in chloroform solutions;

^b)^ Films prepared by spin‐coating on a glass substrate;

^c)^ The absorption edge of the thin film;

^d)^ *E*_g_^opt^ = 1240/*λ*_onset_ (eV);

^e)^ *E*_HOMO_ = −5.1 − (*E*_ox_ − *E*_1/2_ (Fc/Fc^+^));

^f)^ *E*_LUMO_ = *E*_g_^opt^ − *E*_HOMO_;

^g)^ Hole mobility estimated from SCLC.

Cyclic voltammograms (CVs) were performed in acetonitrile to study the electrochemical properties of C‐Cz, as depicted in Figure 1e. The HOMO energy level (*E*_HOMO_) was estimated from the half‐wave oxidation potential of the first oxidation peak, considering the ferrocenium/ferrocene couple at −5.1 eV versus vacuum.^[^ ^47^ ^]^ The first oxidation peak of C‐Cz and spiro‐OMeTAD is located at 0.88 and 0.37 V versus Ag/AgCl, respectively (Figure S6, Supporting Information). Thus, the estimated values of *E*_HOMO_ are −5.48 eV for C‐Cz, and −4.97 eV for spiro‐OMeTAD. This makes C‐Cz an efficient hole extraction/transport mediator between the perovskite layer (VBM = −5.65 eV) and spiro‐OMeTAD owing to their well‐matched energy levels alignment (Figure 1f). Based on the values of *E*_HOMO_ and corresponding *E*_g_^opt^, the LUMO energy level (*E*_LUMO_) of C‐Cz is calculated to be −2.02 eV, indicating that it can effectively block electron transfer at the perovskite/HTM interface to inhibit charge recombination. From the perspective of energy level matching, C‐Cz is capable of extracting holes and blocking electrons in the PSCs, highlighting its potential as an interfacial modification layer.

The charge‐transporting capability of the pristine film of C‐Cz was evaluated by using space‐charge‐limited current (SCLC) measurement.^[^ ^48^ ^]^ The hole‐only device was fabricated with the configuration of an indium‐tin‐oxide (ITO)/poly(3,4‐ethylenedioxythiophene)–polystyrene sulfonate (PEDOT:PSS)/C‐Cz/MoO_3_/Ag to characterize the hole mobility (µ_h_) of C‐Cz. As shown in Figure S7, Supporting Information, µ_h_ of the pristine C‐Cz film was estimated to be 1.45 × 10^−5^ cm^2^ V^−1^s^−1^. It is obvious that the incorporation of carbazole units endows cellulose with electrical conductivity. The proper µ_h_ suggests that C‐Cz can be utilized as *p*‐type semiconductors. In addition, the *π*–*π* stacking of C‐Cz film for efficient charge extraction was investigated by grazing‐incidence wide‐angle X‐ray scattering (GIWAXS) measurements (Figure S8, Supporting Information). The line‐cut profiles in Figure S8a, Supporting Information, show that C‐Cz film exhibits a *π*–*π* stacking diffraction peak at *q* ≈ 1.79 Å^−1^, forming a random orientation, which generally is involved for polymer materials.^[^ ^49^, ^50^, ^51^, ^52^ ^]^ The calculated *π*–*π* stacking distance of C‐Cz is 3.5 Å, suggesting that the introduction of carbazole units can enhance intermolecular interactions and tighten *π*–*π* stacking, resulting in efficient charge extraction and transport when applied as an interlayer in PSCs.

**Figure 2:**
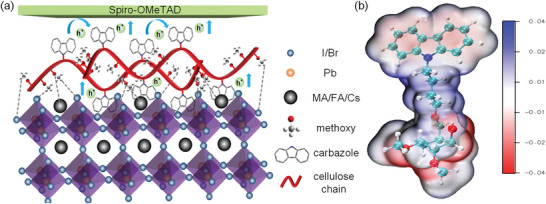
a) Schematic illustration of the interaction between C‐Cz and perovskite. b) Electrostatic surface potential (ESP) maps of C‐Cz monomer obtained by density DFT calculation.

**Figure 3:**
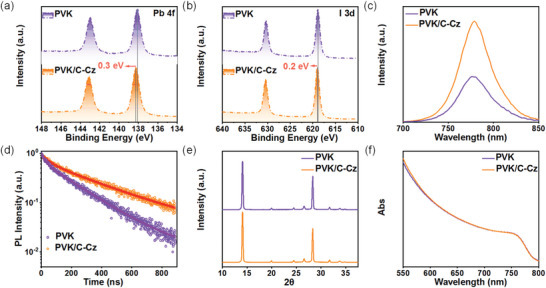
XPS characteristic peaks of a) Pb 4f and b) I 3d of the pure perovskite (PVK) and PVK/C‐Cz films. c) Steady‐state photoluminescence (PL) and d) time‐resolved PL decay spectra of PVK and PVK/C‐Cz films. e) X‐ray diffraction patterns and f) normalized ultraviolet–visible absorption spectra of PVK and PVK/C‐Cz films.

The designed C‐Cz exhibits not only proper energy levels and good hole extraction/transport capabilities but also passivation defects on perovskite surfaces. **Figure** **2**a illustrates the mechanism of interaction between C‐Cz and perovskite. Previous works have shown that the electron lone pairs of oxygen atoms in ether groups can passivate the undercoordinated Pb^2+^ vacancies on the perovskite surface.^[^ ^35^, ^39^ ^]^ Besides, the oxygen and hydrogen atoms in methoxy groups can adsorb to the perovskite surface via the interactions with methylammonium cation and iodide ion.^[^ ^34^, ^40^, ^53^, ^54^ ^]^ To prove the interaction mechanism mentioned above, the electrostatic surface potential (ESP) was calculated to study the static charge distribution using the C‐Cz monomer. As shown in Figure 2b, the negative partial charges are delocalized on the oxygen atoms in methoxy and ether groups, which is anticipated to passivate the undercoordinated Pb^2+^ on the perovskite surface. To confirm the bonding interactions between C‐Cz and lead ions, we applied FTIR spectra to investigate the C—O stretch (Figure S9, Supporting Information). The stretching vibration of the C—O bond in C‐Cz was located at 1077 cm^−1^ and it shifted to 1091 cm^−1^ (*∆* = 14 cm^−1^) upon interaction with PbI_2_. Such a shift in the C‐Cz molecule resulted from the delocalization of electrons in oxygen to form Lewis acid–base adduct with PbI_2_.^[^ ^15^, ^55^ ^]^ X‐ray photoelectron spectroscopy (XPS) measurements were performed further to investigate the interaction between C‐Cz and the perovskite layer (**Figure** **3**a,b). The characteristic peaks of the Pb 4f and I 3d spectrum shift toward the higher binding energy after treating with C‐Cz (0.3 eV for Pb 4f and 0.2 eV for I 3d, respectively), supporting the strong electronic interaction between C‐Cz and Pb and I on the perovskite surface, and further suggesting that C‐Cz effectively passivates the perovskite surface.^[^ ^13^, ^16^, ^56^, ^57^ ^]^

To directly investigate the passivation effect and charge extraction capability of C‐Cz, steady‐state photoluminescence (PL) and time‐resolved PL (TRPL) measurements were carried out. The C‐Cz modified perovskite film exhibits a dramatically enhanced PL intensity than the pristine film (Figure 3c), indicating that the suppressed non‐radiative charge recombination mainly occurs at the defects.^[^ ^12^ ^]^ Fitted TRPL decay provides insights into the charge carrier dynamics, which was fitted using an empirical biexponential equation.^[^ ^58^, ^59^, ^60^ ^]^ The fitted parameters are summarized in Table S3, Supporting Information. The TRPL spectra further confirmed the results of the steady‐state PL (Figure 3d). The average lifetime (*τ*_avg_) of the C‐Cz modified film (344.69 ns) is significantly longer than that of the pristine films (206.09 ns). The slower decay generally indicates fewer defects in the perovskites and thus suppressed non‐radiative recombination of charge carriers, leading to a strong PL intensity in the spectrum.^[^ ^12^ ^]^ These results are in good agreement with the FTIR and XPS analysis. Based on the above, we concluded that the perovskites with C‐Cz have excellent defect passivation due to the strong interaction between C‐Cz and Pb^2+^/I^−^ in the perovskites.

In order to further study the effect of interfacial modification on the basic properties of the perovskite films, the X‐ray diffraction (XRD) patterns and UV–vis absorption spectra of the perovskite films with or without C‐Cz were collected. As shown in Figure 3e, all the films exhibit similar cubic phase diffraction peaks. The main peaks at 14.1° and 28.3° correspond to the (001) and (002) crystal planes, respectively.^[^ ^61^ ^]^ The similar XRD spectra confirm that the existence of C‐Cz does not affect the main crystal structure of perovskite. Figure 3f shows that C‐Cz modification does not influence the light‐harvesting property of the perovskite films, indicating that the band gap of perovskite is maintained. In addition, GIWAXS measurements were also carried out to obtain more information about the crystal structure of the perovskite films (Figure S10, Supporting Information). Compared with the pristine film, no new diffraction pattern appeared for the C‐Cz modified perovskite film, which is consistent with the XRD result. This might be because the interlayer with C‐Cz is too thin to be detected by both XRD and GIWAXS measurements.^[^ ^62^ ^]^

To investigate the influence of surface modification on the perovskite morphology changes, scanning electron microscopy (SEM) and atomic force microscopy (AFM) measurements were performed (Figure S11, Supporting Information). In the pristine perovskite film, the dark perovskite grains are surrounded by some white PbI_2_ grains, which is consistent with reported results (Figure S11a,b, Supporting Information).^[^ ^63^, ^64^ ^]^ After coating with a thin layer of C‐Cz, the PbI_2_ grains are fully covered, while the SEM images become misty due to relatively less conductivity of the surface modification layer than that of perovskite.^[^ ^63^ ^]^ Moreover, the root‐mean‐square (RMS) roughness of perovskite/C‐Cz film is reduced to 14.9 nm compared to 18.1 nm of the pristine perovskite film (Figure S11c,d, Supporting Information). The significantly decreased surface roughness of C‐Cz leads to excellent contact between perovskite and hole transporting layer, thus reducing undesired shunt losses in PSC devices. In addition, the existence of C‐Cz on the perovskite surface was confirmed by the energy dispersive X‐ray spectroscopy (EDX). Relevant elemental mappings of the C‐Cz‐modified perovskite film are shown in Figure S12, Supporting Information. The characteristic element of C‐Cz is oxygen (O). The presence of the element O reveals that the C‐Cz covers the perovskite surface. It tends to form a uniform interface between the perovskite and HTM.

**Figure 4:**
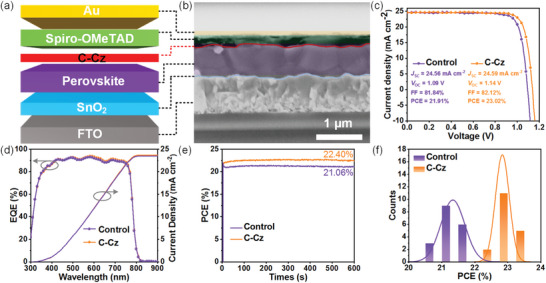
a) Device architecture of PSC. b) Cross‐section SEM image of the PSC with the C‐Cz interlayer. c) *J–V* curves of the champion PSCs with and without C‐Cz. d) EQE spectra with the integrated *J*_SC_ and e) steady‐state power outputs of the champion PSCs with and without C‐Cz. f) Histogram of PCE distribution.

**Figure 5:**
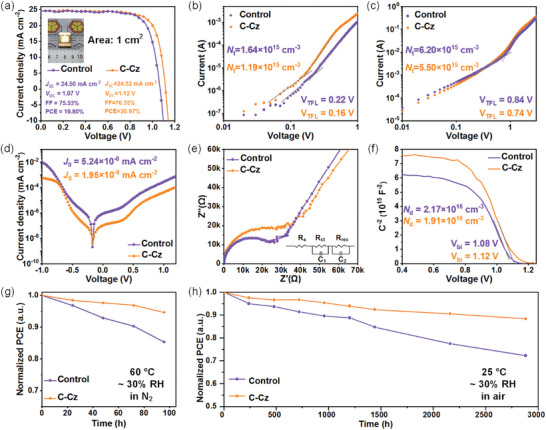
a) *J*–*V* curves of the champion large‐area PSCs (active area of 1 cm^2^) with and without C‐Cz. Different *J*–*V* curves of b) hole‐only (device configuration: ITO/PEDOT:PSS/perovskite/interfacial layer/ spiro‐OMeTAD/Ag) and c) electron‐only devices (device configuration: ITO/SnO_2_/perovskite/interfacial layer/PCBM/BCP/Ag) in the dark. d) Dark *J*–*V* curves of different perovskite devices. e) Nyquist plots of PSCs with and without interfacial layers. f) Mott–Schottky plots of the corresponding PSCs. PCE evolution of the unencapsulated PSCs without and with C‐Cz under g) N_2_ at 60 °C and h) ambient atmosphere at room temperature (RH = 30 ± 5%).

In order to demonstrate the interfacial effect of C‐Cz, we fabricated a series of planar PSCs with an n‐i‐p configuration of FTO/SnO_2_/perovskite/C‐Cz/spiro‐OMeTAD/Au using a one‐step method (**Figure** **4**a). The thickness of C‐Cz films was optimized by changing the solution concentration, and the optimized concentration of champion performance is 1 mg mL^−1^ in chloroform solution (Figure S13, Supporting Information). The representative cross‐sectional SEM image of the C‐Cz‐based device is displayed in Figure 4b. A thin interfacial layer of C‐Cz (3.6 nm) was inserted between the perovskite film and spiro‐OMeTAD. Figure 4c displays the current density–voltage (*J–V*) curves of the champion devices without and with an interfacial layer under the standard AM 1.5 G illumination. The C‐Cz‐based device achieves the highest PCE of 23.02% with a short‐circuit current (*J*_SC_) of 24.59 mA cm^−2^, *V*_OC_ of 1.14 V and FF of 82.12%, which is among the highest performance of PSCs based on one‐step method (Figure S14, Supporting Information). In contrast, the control device exhibits a much inferior PCE of 21.91% owing to the lower *V*_OC_ of 1.09 V and FF of 81.84%. The significantly boosted photovoltaic performance of the C‐Cz‐based device could be attributed to the combined effect of defect passivation and improved interfacial charge extraction. Compared to the pristine device, the C‐Cz‐based device shows higher external quantum efficiency (EQE) over the whole light‐absorption range (Figure 4d). The integrated *J*_SC_ is 23.50 and 23.69 mA cm^−2^ for the control and C‐Cz‐based device, respectively, which matches well with the results from the *J–V* curves. Moreover, the efficiencies of PSCs were further verified by the steady‐state power output at the maximum power point (MPP), and stabilized PCEs of 22.40% and 21.06% were found for the cells with and without C‐Cz over 600 s, respectively (Figure 4e). In addition, the C‐Cz‐based cells exhibit good repeatability with an average PCE (PCE_avg_) of 22.83%, much better than the PCE_avg_ (21.33%) of the control devices (Figure 4f). To evaluate the scalability of the interfacial layer, we further fabricated PSCs with an active area of 1 cm^2^ (**Figure** **5**a). The optimized large‐area device based on C‐Cz also yields a higher PCE of 20.97% with significantly enhanced FF (76.35%) and *V*_OC_ (1.12 V), whereas the control device shows a PCE of only 19.81% with FF of 75.53% and *V*_OC_ of 1.07 V.

To better understand the increased performance of the modified PSCs, we measured the hole‐only (ITO/PEDOT:PSS/perovskite/interfacial layer/spiro‐OMeTAD/Ag) and electron‐only device (ITO/SnO_2_/perovskite/interfacial layer/PCBM/BCP/Ag) to evaluate the surface defect density (*N*_t_) of the perovskite film using the following equation:^[^ ^65^ ^]^

(1) $$N_{t}=\;\frac{2\varepsilon_{0}\varepsilon_{r}V_{\mathrm{TFL}}}{eL^{2}}$$

where *ε*_0_ is the vacuum permittivity, *ε*_r_ is the relative dielectric constant, the transition voltage between ohmic and trap‐filling regions, *e* is the electron charge, and *L* is the perovskite film thickness. As shown in Figure 5b, the *V*_TFL_ for the perovskite with and without C‐Cz is 0.16 and 0.22 V, and the corresponding hole trap densities are determined to be 1.19 × 10^15^ and 1.64 × 10^15^ cm^−3^, respectively. On the other hand, the electron trap densities are 5.50 × 10^15^ and 6.20 × 10^15^ cm^−3^ with 0.74 and 0.84 V, respectively (Figure 5c). The *N*_t_ of the perovskite film coated with C‐Cz is much decreased, suggesting that C‐Cz can effectively passivate the defects on the perovskite surface. The reduction of defects has a positive effect on enhancing *V*_OC_ and suggests that C‐Cz plays a crucial role in reducing recombination centers. Moreover, to reveal the recombination pathway, we then investigated the *V*_OC_ loss mechanism at the device level. *V*_OC_ can be expressed as in the following equation:^[^ ^66^ ^]^

(2) $$V_{\mathrm{OC}}=\;\frac{mk_{B}T}{e}\ln\left(\frac{J_{\mathrm{SC}}}{J_{0}}+1\right)$$

where *m* is the ideal factor used to evaluate the dominating recombination mechanism, *J*_0_ and *J*_SC_ represent the reverse‐bias saturation current density and the reverse‐bias saturation photocurrent density in the dark, respectively. The smaller *J*_0_ manifests that lower shallow defect states exist in the devices. As shown in Figure 5d, the procured *J*_0_ of the C‐Cz‐based PSCs (1.95 × 10^−9^ mA cm^−2^) was much smaller than that of the control devices (5.24 × 10^−8^ mA cm^−2^), indicating that the trap‐assisted Shockley–Read–Hall recombination was suppressed by capping with C‐Cz. In addition, electrochemical impedance spectroscopy (EIS) was adopted to evaluate the recombination behavior further. Figure 5e compares the Nyquist plots of PSCs with or without C‐Cz in dark conditions. The high‐frequency region is usually dominated by charge transfer resistance (*R*_ct_), and the low‐frequency region is related to the recombination resistance (*R*_rec_).^[^ ^12^, ^60^ ^]^ The PSC with C‐Cz shows a considerably decreased *R*_ct_ (from 9.79 to 6.40 Ω cm^−2^) and increased *R*_rec_ (from 27.64 to 38.96 KΩ cm^−2^), further confirming the facilitated charge transfer and suppressed carrier recombination at the perovskite/C‐Cz interface. These results conform with the analysis of XPS, PL, and TRPL mentioned above.

Mott–Schottky analysis was further conducted to estimate the built‐in potential and the driving force for photo‐generated carriers. The built‐in potential and charge distribution at the perovskite/C‐Cz interface can be obtained from the Mott–Schottky equation:^[^ ^67^ ^]^

(3) $$\frac{1}{C^{2}}=\frac{2}{A^{2}e\varepsilon_{0}\varepsilon N_{d}}\;\left(V_{\mathrm{bi}}-V\right)$$

where *C* is the capacitance of the space charge region, A is the active area, *ε* is the relative dielectric constant of perovskite, *ε*_0_ is the vacuum permittivity (8.85 × 10^−12^ F m^−1^), *V*_bi_ is the built‐in potential, *V* is the applied voltage, and *N*_d_ is the carrier density, respectively. As shown in Figure 5f, the *V*_bi_s are 1.08 and 1.12 V for the control and C‐Cz‐based devices, respectively. The increased *V*_bi_ implies an improved driving force for the separation of photo‐generated charge carriers, which agrees well with the trend of *V*_OC_ values derived from *J*–*V* curves. Meanwhile, the *N*_d_ of control and C‐Cz‐based devices are calculated to be 2.17 × 10^16^ and 1.91 × 10^16^ cm^−3^, respectively. The decreased carrier density indicates less carrier accumulation at the perovskite/C‐Cz interface. The results are in accordance with the measurements of electrochemical impedance spectroscopy (EIS). These data consistently suggest that C‐Cz acts as a bifunctional interlayer for both defect passivation and carrier extraction and transfer.

Stability issues remain the main obstacle to the commercialization of PSCs since the perovskite films show high sensitivity toward humidity, oxygen, and heat. Thus, the long‐term stability was tested to compare the environmental durability of the control and C‐Cz‐based devices. The wetting property was studied by water contact angle (CA) measurements. As shown in Figure S15a,b, Supporting Information, the CA of the bare perovskite increases distinctly from 32.7° to 68.7° when it is modified with C‐Cz, which can hinder the water penetration into the perovskite interface, thus mitigating the perovskite degradation. On the other hand, the CA of the perovskite modified with C‐Cz is slightly larger than that of doped spiro‐OMeTAD (60.9°), proving that C‐Cz can add up the hydrophobicity of perovskite surface (Figure S15c, Supporting Information). In addition, more surface defects may lead to more rapid degradation of perovskite in a humid environment.^[^ ^68^ ^]^ The C‐Cz material can reduce the surface defects of the perovskite film, as proven by FTIR, XPS, PL, TRPL, etc., thus reducing the surface defects of perovskite film. Therefore, the moisture stability can be further enhanced by involving C‐Cz between perovskite and doped spiro‐OMeTAD.

Hence, the long‐term stability of the unencapsulated devices is investigated with a relative humidity (RH) of 30 ± 5%. As illustrated in Figure S16, Supporting Information, the C‐Cz‐based device still keeps over 92% of the initial efficiency after 160 days of storage in the N_2_ glove box, whereas the control device decreases to 80% of the initial PCE value. The thermal stability of the devices was further evaluated under continuous heating at 60 °C in an N_2_ atmosphere (Figure 5g). The PCE of the control device lost ≈15% after 96 h, while the C‐Cz‐based device lost only about 6% of its initial PCE. Moreover, we further tested the long‐term stability of the unencapsulated devices under an ambient environment with a relative humidity (RH) of 30 ± 5% at room temperature (Figure 5h). Encouragingly, the C‐Cz‐based device maintained over 88% of its original PCE after 2800 h. In contrast, the control device suffered from a rapid decline to only 72% of the initial efficiency. Considering other functional layers in PSCs devices are the same, the above‐improved long‐term and thermal stabilities of the C‐Cz‐based device can be ascribed to decreased defect density and enhanced moisture‐resistance of perovskite film.

## 3 Conclusion

In summary, we developed a low‐cost cellulose derivative as a bifunctional interfacial material to facilitate charge extraction/transport and improve the stability of PSCs. The presence of C‐Cz effectively reduced the trap states and hence significantly suppressed non‐radiative recombination on the surface and in the bulk of the perovskite film. Meanwhile, the dangling carbazole groups were demonstrated to stabilize the HOMO energy level and promote hole transport, further improving hole extraction at the perovskite/HTM interface. As a result of this synergetic effect, the PSC based on C‐Cz achieved the best PCE of 23.02% compared with 21.91% of the pristine device. More encouragingly, the long‐term and thermal stabilities of the C‐Cz‐based PSCs are greatly improved, which are credited to the increased hydrophobicity and reduced defect density, effectively preventing the perovskite film from moisture penetration and mitigating perovskite degradation. Our work reveals the importance of the functional group configuration in the cellulose derivative in boosting the performance and stability of PSCs and provides a powerful strategy for designing low‐cost and bifunctional cellulose‐based material for the same purpose.

## 4 Experimental Section

Experimental details are provided in the Supporting Information section.

## Conflict of Interest

The authors declare no conflict of interest.

## Supporting information

Supporting Information

## Data Availability

The data that support the findings of this study are available in the supplementary material of this article.
