# Supplementary material for: Bifunctional Cellulose Interlayer Enabled Efficient Perovskite Solar Cells with Simultaneously Enhanced Efficiency and Stability
Source: Adv Sci (Weinh). 2023 Feb 7;10(8):2207202. doi: 10.1002/advs.202207202 (PMC10015901; doi:10.1002/advs.202207202)
Supplement: Supplementary file 1 — Supporting Information [file ADVS-10-2207202-s001.pdf]

Supporting Information

Bifunctional Cellulose Interlayer Enabled Efficient Perovskite Solar Cells with Simultaneously Enhanced Efficiency and Stability

Zilong Zhang, Can Wang, Feng Li, Lusheng Liang, Liulian Huang, Lihui Chen, Yonghao Ni, Peng Gao, and Hui Wu**

1. Experimental Section

1.1 Materials and Synthesis.

All reaction reagents were purchased from commercial sources (Sigma-Aldrich, TCI, J&K, Aladdin and Innochem, etc.) and are used without further purification. Solvents were purified by standard methods and dried if necessary. The SnO_2 colloid precursor was obtained from Alfa Aesar. Lead iodide (PbI_2), lead bromide ($PbBr_2$) and cesium iodide (CsI) were purchased from Sigma-Aldrich. Formamidinium iodide (FAI) and methylammonium bromide (MABr) were synthesized by reacting formamidine acetate (Aladdin) and hydroiodic acid (55.0-58.0 wt% in H_2O, Aladdin), methylamine (30-33 wt% in ethanol, Aladdin) and hydrobromic acid (48 wt% in H_2O, Aladdin), respectively. Spiro-OMeTAD was purchased from Derthon Optoelectronic Materials Science Technology Co LTD. The anhydrous solvents were purchased from Sigma-Aldrich. Cellulose was extracted from a pinus massoniana dissolving pulp board, supplied by Fujian Qingshan Paper Industry Co., Ltd., China. 9-(4-bromobutyl)-9H-carbazole was synthesized according to previous literature procedures.[1] The synthetic routes to **C-Cz** was illustrated in **Scheme S1**. The preparation details were described as follows.

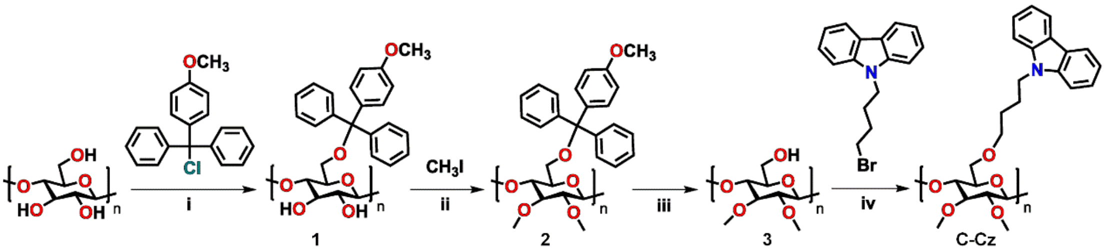

Scheme S1. Synthetic routes to **C-Cz**. Reagents and conditions: (i) DMAc:LiCl, 4-methoxytriphenylmethyl chloride, pyridine, 80 °C, 8 h; (ii) CH_3I, NaH, DMSO, 70 °C, 24 h;

(iii) THF, concentrated HCl, rt, 24 h; (iv) 9-(4-bromobutyl)-9H-carbazole, DMF, NaH, tetra-*n*-butylammonium iodide, 60 °C, 48 h.

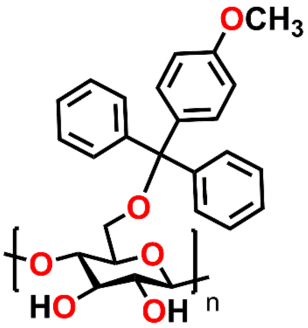

Synthesis of 6-O-p-methoxytrityl cellulose (1). 4-methoxytriphenylmethyl chloride (28.6 g, 92.5 mmol) was added to a solution of cellulose (5 g), LiCl (7.5 g), pyridine (11.2 mL, 4.5 mol per AGU), and DMAc (150 mL), and the reaction mixture was stirred at 80 °C for 8 h. After cooling to room temperature, the resulting mixture was poured in to methanol. The precipitates were filtered, washed with methanol, and dried under vacuum to give **1** (11.3 g, 90%).

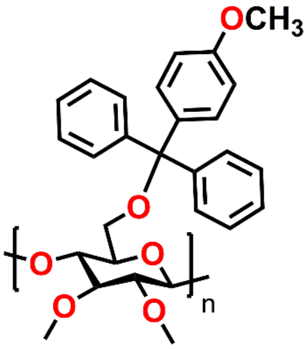

Synthesis of 6 -O- p-methoxytrityl-2,3 -di-O-methylcellulose (2). 5 g **1** was added to a solution of 300 mL DMSO, sodium hydride (4.7 g, 0.16 mol). After stirring at room temperature for 1 h, methyl iodide (4.68 mL) was added. Further additions of methyl iodide followed after 2, 3, and 4 h (0.78 mL each case, total 7.08 mL, 16.14 g, 0.113 mol). The mixture was allowed to react for 24 h at 70 °C. The product was precipitated in methanol, washed with methanol, and dried under vacuum to give **2** (4.1 g, 80%).

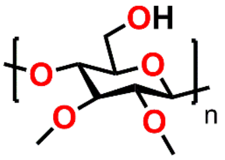

Synthesis of 2,3 -di-O-methylcellulose (3). **2** (2g) was dissolved in THF (100 mL) and concentrated HCl (2 mL) was added. After stirring at room temperature for 24 h, the product

was precipitated into acetone, washed with acetone, and dried under vacuum to give **3** (0.48 g, 63%).

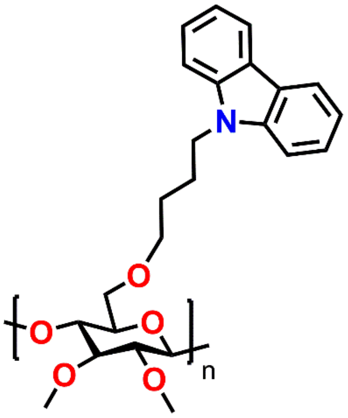

Synthesis of 6-O-[4-(9H-carbazol-9-yl)butyl]- 2,3-di-O-methyl Cellulose (C-Cz). To a DMF solution of **3** (0.089 g, 0.47 mmol), sodium hydride (0.19 g, 4.7 mmol) was added. After 30 min, 9-(4-bromobutyl)-9H-carbazole (0.43 g, 1.4 mmol) and tetra-*n*-butylammonium iodide (52 mg, 0.14 mmol) were added at room temperature. The mixture was stirred at 60 °C for 48 h. The reaction mixture was poured into acetone. The precipitated was washed with acetone, methanol, and dried under vacuum to give **C-Cz** (0.14 g, 73%). ^{1}H NMR (500 MHZ, $CDCl_3$) δ: 8.08 (m, 2H), 7.48-7.35 (m, 4H), 7.22 (m, 2H), 4.44-3.82 (m, 3H), 3.72-3.16 (m, 13H), 2.94 (m, 1H), 1.92-1.58 (m, 4H). ^{13}C NMR (500 MHZ, $CDCl_3$) δ: 140.39, 125.66, 122.81, 120.34, 118.78, 108.67, 103.16, 85.28, 71.23, 60.57, 42.82, 29.72, 27.44, 26.07. M_w = 79 kDa.

1.2 Theoretical Calculations.

The ground-state geometry optimization was calculated using density functional theory (DFT) method at the B3LYP/6-31G(d,p) level of theory with the Gaussian 09 program package.[2] Stationary points were verified by frequency analysis. The optimized structures were found to be stable. The calculated molecular electronic static potential (ESP) results were obtained with the Multiwfn 3.7 program.[3]

1.3 Characterization and Measurements.

The ^{1}H NMR and ^{13}C NMR spectra were performed on the Bruker AVANCE III 500 MHz spectrometer. Thermogravimetric (TGA) measurements were carried out on a Mettler Toledo apparatus at a heat ramp of 10 °C min^{-1} under nitrogen. Ultroviolet-visble (UV-Vis) absorption spectra were recorded on a Cary 5000 spectrophotometer. Cyclic voltammetry measurements were carried out under nitrogen atmosphere using the CHI760E voltammetric workstation with a N_2-saturated solution of 0.1 M tetra-*n*-butyl ammonium hexafluoro phosphate (Bu_4NPF_6) in acetonitrile as the supporting electrolyte. A glassy carbon working electrode, a platinum wire counter electrode, and a silver chloride (Ag/AgCl) reference

electrode were employed, and the ferrocene/ferrocenium redox couple (Fc/Fc^+) was used as the reference for all measurements with a scanning rate of 100 mV s^{-1}. Atomic force microscopy (AFM) measurements were conducted using a Bruker Multimode-8J microscope in air mode and ScanAsyst software. Scanning electron microscope (SEM) and energy dispersive X-ray spectroscopy (EDX) were measured by a SUPRA 55, Zeiss, Germany, operated at an acceleration voltage of 5 kV. The thickness of the film spin coated on c-Si substrates by spectroscopic ellipsometry (HORIBA Detection Head). XRD measurements were performed using a SmartLab (Rigaku) system equipped with a Cu-Kα source. The X-ray photoelectron spectrum (XPS) was performed using an X-ray photoelectron spectroscopy system (Axis Supra, Shimadzu). The grazing incident wide-angle X-ray scattering (GIWAXS) measurements were performed at BL19B2 beamline of SPring-8. The sample was irradiated with an X-ray energy of 12.39 keV (λ= 1 Å) at a fixed-incident angle on the order of 0.12° through a Huber diffractometer. The GIWAXS pattern was recorded with a two-dimensional image detector (Pilatus 300 K). Steady-state photoluminescence was carried out with a fluorescence spectrophotometer (FLS980, Edinburgh Instruments). Current density-voltage (*J*-*V*) characteristics were performed on a source meter (Keithley 2401) under 100 mW cm^{-2} simulated AM 1.5 G irradiation with a solar simulator (Enli Tech, Taiwan). The active area of devices is 0.1 cm^2. The external quantum efficiency (EQE) spectrum was performed on QE-R 3011, Enli at DC mode. Mott-Schottky measurements were tested using an electrochemical workstation (Zennium Zahner, Germany). Electrochemical impedance spectroscopy (EIS) measurements were carried out under dark at applied voltages (0.6 V) using an electrochemical workstation (Zennium Zahner, Germany) with an AC perturbation of 10 mV ranging from 100 mHz to 1 MHz. Space-charge limited current (SCLC) measurement was performed on a Keithley 2401 source meter ranging from 0 V to 3 V. The hole mobility (μ) can be extracted from the trap-free SCLC regime, according to Mott-Gurney law:[4]

$$J_D = \frac{9}{8}\varepsilon_0\varepsilon_r\mu\frac{V^2}{d^3} \qquad (1)$$

Where J_D is the current density, V_b is applied voltage, ε_r is the relative dielectric constant of organic materials ($\varepsilon_r = 3$), ε_0 is the vacuum permittivity, and q is the electron charge.

1.4 Device Fabrication.

The glass substrates (AGC Company) including FTO layer were chemically etched, cleaned with deionized water, acetone, and anhydrous ethanol for 10 min, respectively. Then, the clean substrates were dried by N_2. The substrates were treated by plasma for 15 min. Subsequently a thin layer of SnO_2 nanoparticle film (SnO_2 colloid precursor diluted by deionized water, 1:3, weight ratio) were spin-coated on the FTO substrates at 3000 rpm for 30

s and annealed in ambient air at 150 °C for 30 min. $(FAPbI_3)_{0.92}(MAPbBr_3)_{0.08}$ perovskite precursor solution was prepared by dissolving FAI, MABr, MACl, PbI_2, and $PbBr_2$ in a mixed solvent (DMF: DMSO = 9:1). After stirring for 30 min, 57.5 μL CsI which was previously dissolved as a 1.5 M stock solution in DMSO was added to the mixed perovskite precursor solution. Then, the perovskite precursor solution was spin-coated onto the substrates with SnO_2 via using a one step procedure. Perovskite precursor solution was spin-coated on FTO/SnO_2 sample at 1000 rpm for 10 s and then 5000 rpm for 25 s. 160 µL of chlorobenzene was dropped at the last 5 s, and then the sample was annealed at 150 ^oC for 10 min. 60 µL of C-Cz solution (0.5-1.5 mg/ml in chloroform solution) was spin-coated onto the perovskite layer at 4000 rpm for 30 s and heated at 100 ^oC for 5 min. Subsequently, 55 µL spiro-OMeTAD solution (72.3 mg spiro-OMeTAD in 1 mL chlorobenzene with additives including 17.5 μL solution of Li-TFSI (520 mg mL^{-1} in acetonitrile) and 28.5 μL of *t*BP was spin-coated on the cooled substrates at 4000 rpm for 30 s. Finally, 70 nm of the gold counter electrode was thermally evaporated under high vacuum.

1.5 Stability Measurement.

The dark long-term stability assessment of the perovskite solar cells was carried out by repeating the *J–V* characterizations over various times. The unsealed devices were stored under ambient atmosphere with 30% relative humidity at room temperature without encapsulation.

2. Additional Figures and Tables

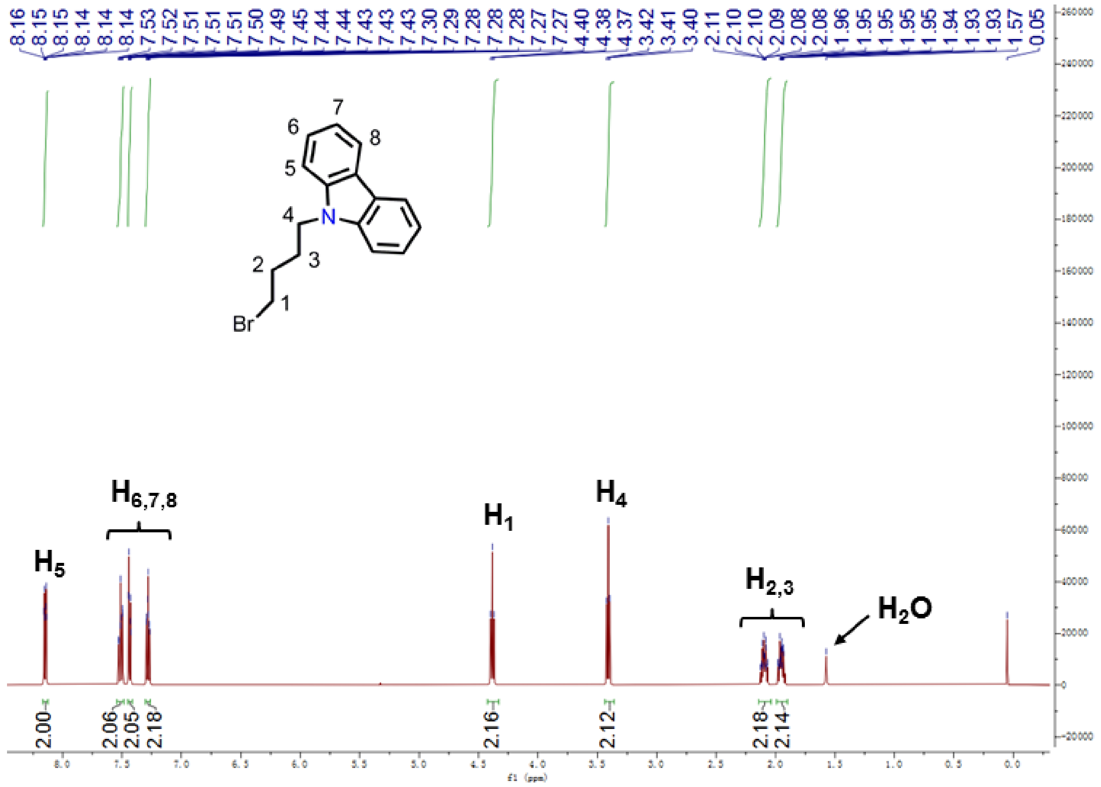

Figure S1. ^{1}H NMR spectra of 9-(4-bromobutyl)-9H-carbazole.

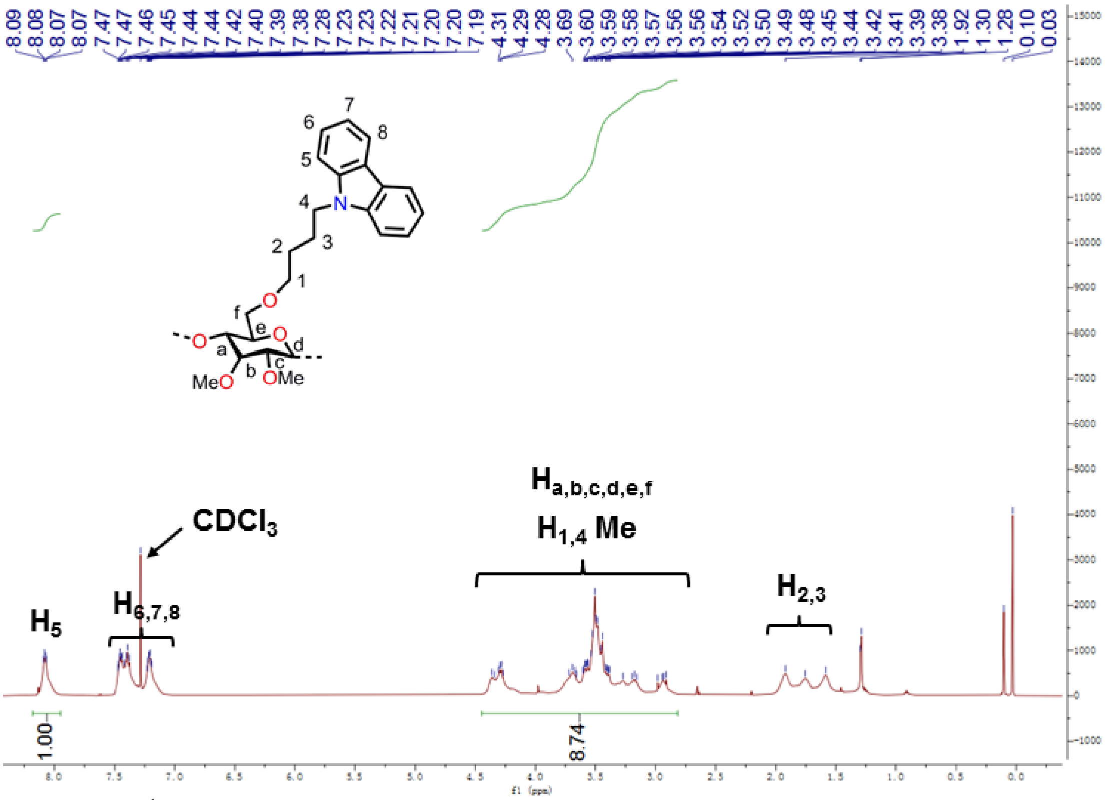

Figure S2. ^{1}H NMR spectra of **C-Cz**.

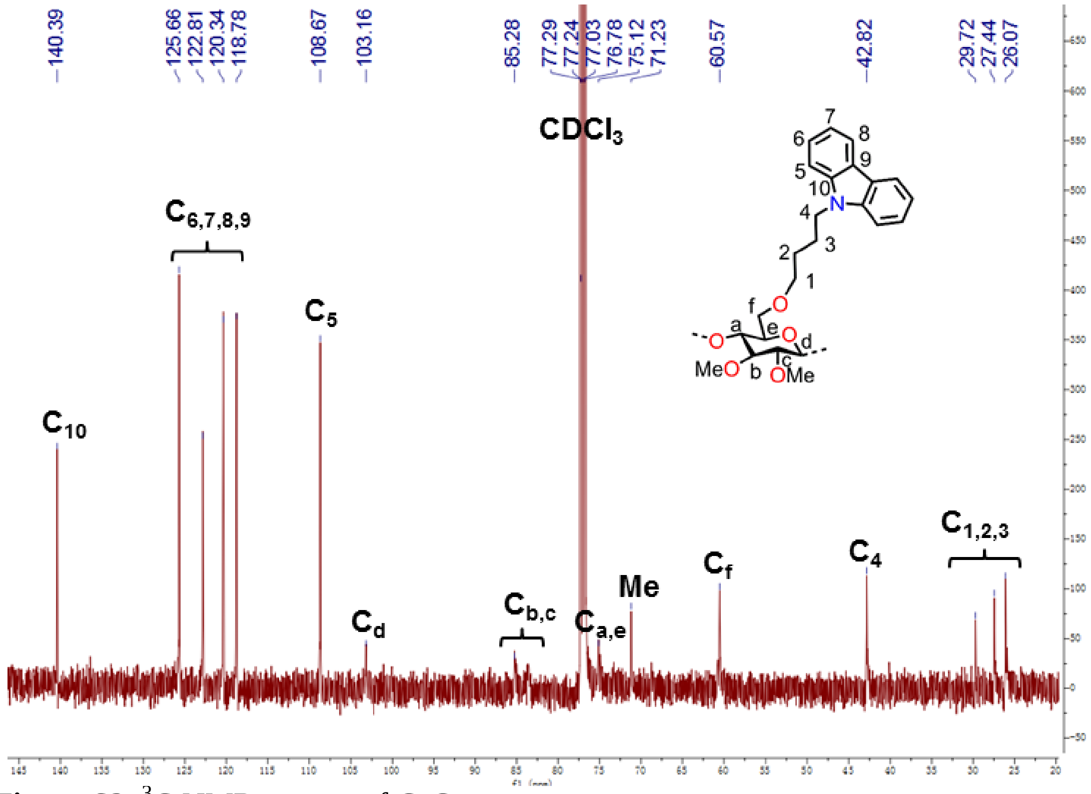

Figure S3. ^{3}C NMR spectra of **C-Cz**.

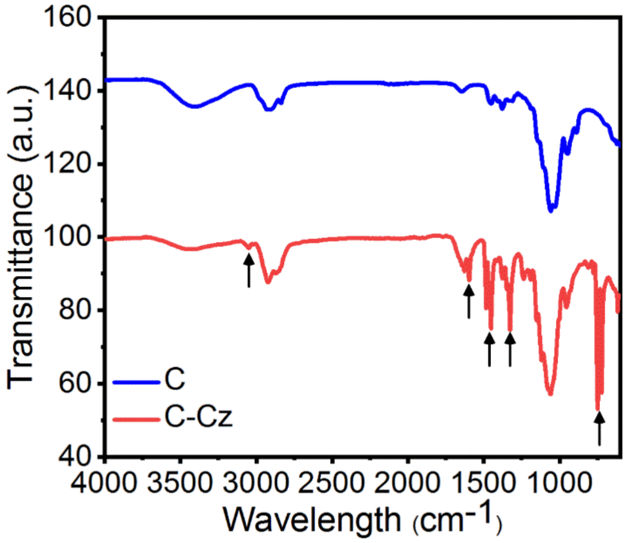

Figure S4. FTIR spectra of cellulose and **C-Cz** (arrows points to the characteristic peaks of the carbazole moieties).

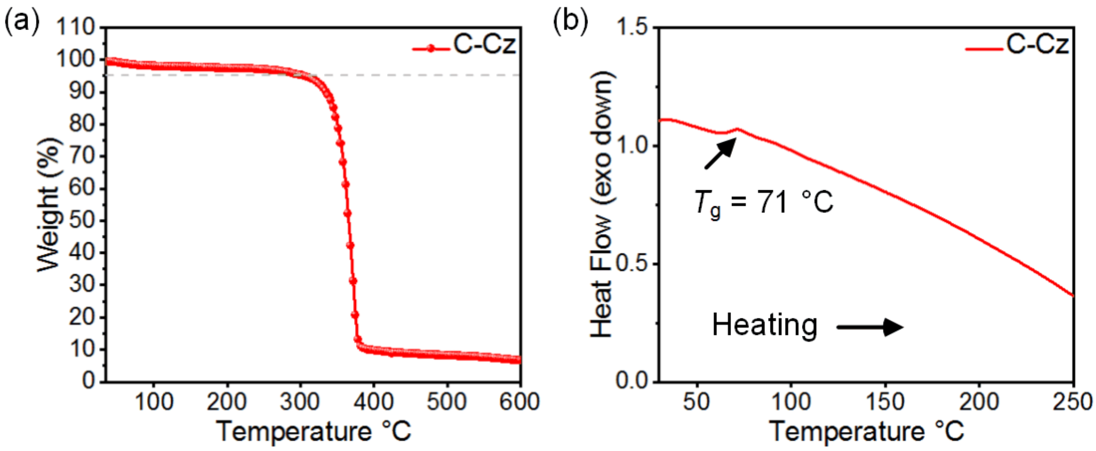

Figure S5. a) TGA and b) DSC curves of **C-Cz**.

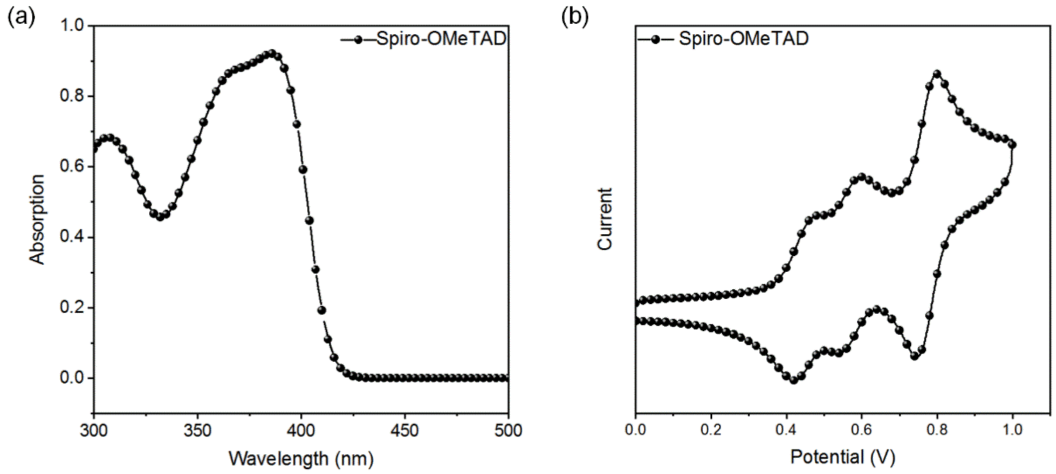

Figure S6. a) Normalized UV−vis absorption and b) cyclic voltammogram (CV) spectra of spiro-OMeTAD in dichloromethane.

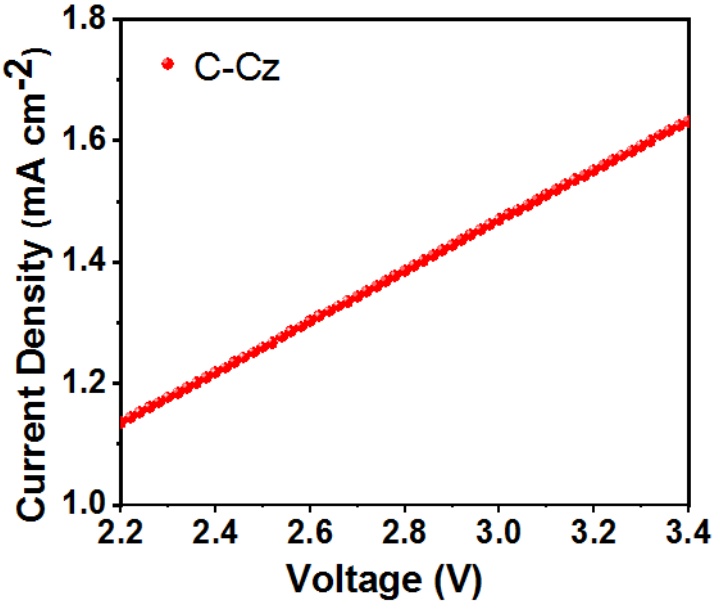

Figure S7. The space-charge limited current (SCLC) measurements of hole only devices with the configuration of ITO/PEDOT:PSS/**C-Cz**/MoO_3/Ag.

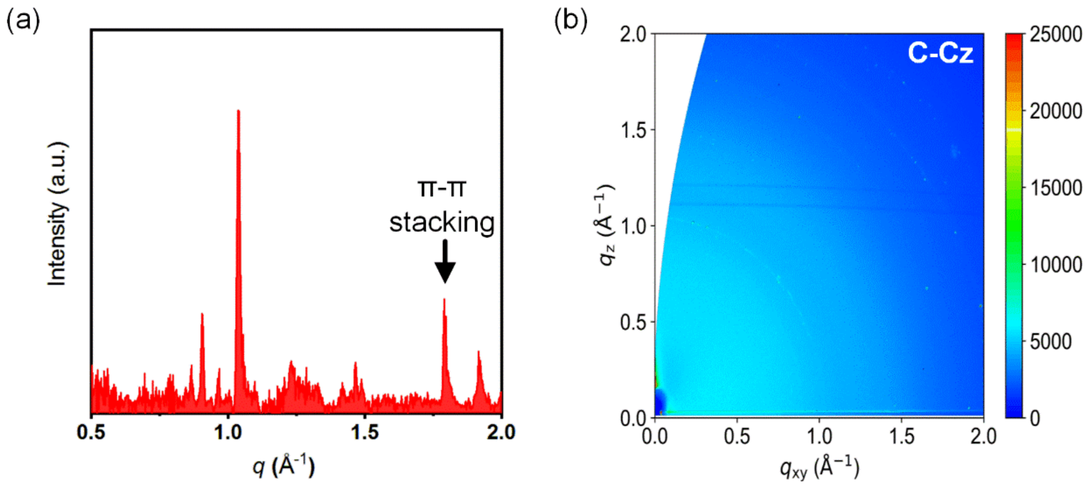

Figure S8. a) The GIWAXS integrated line files and b) GIWAXS pattern of C-Cz film.

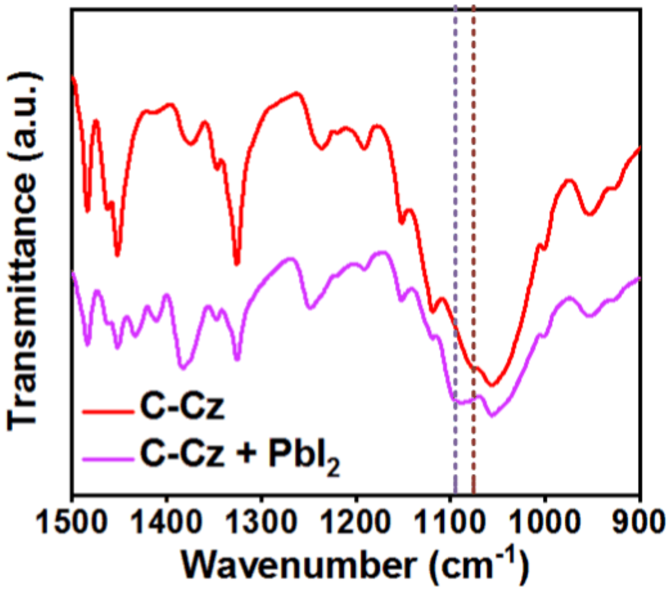

Figure S9. FTIR spectra of C-Cz and C-Cz-PbI_2 films.

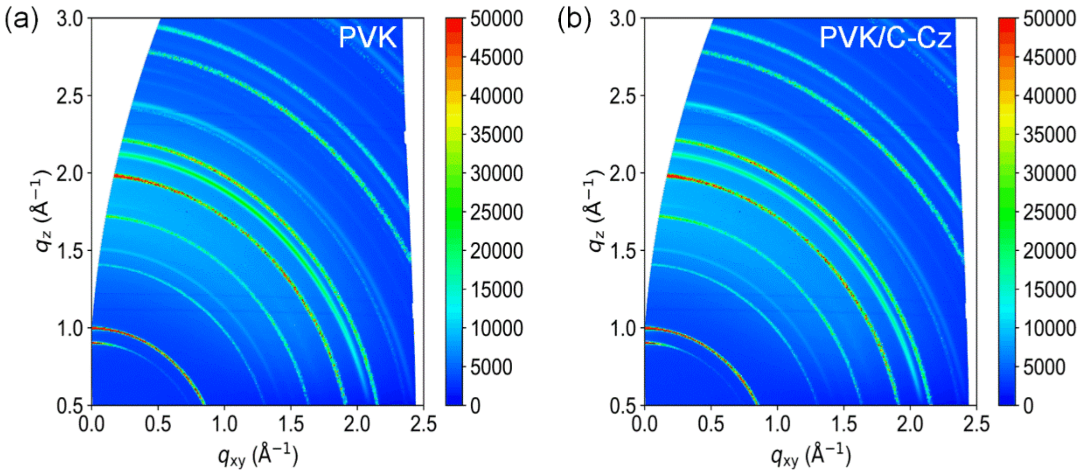

Figure S10. The GIWAXS patterns of a) pristine perovskite (PVK) and b) PVK/C-Cz films.

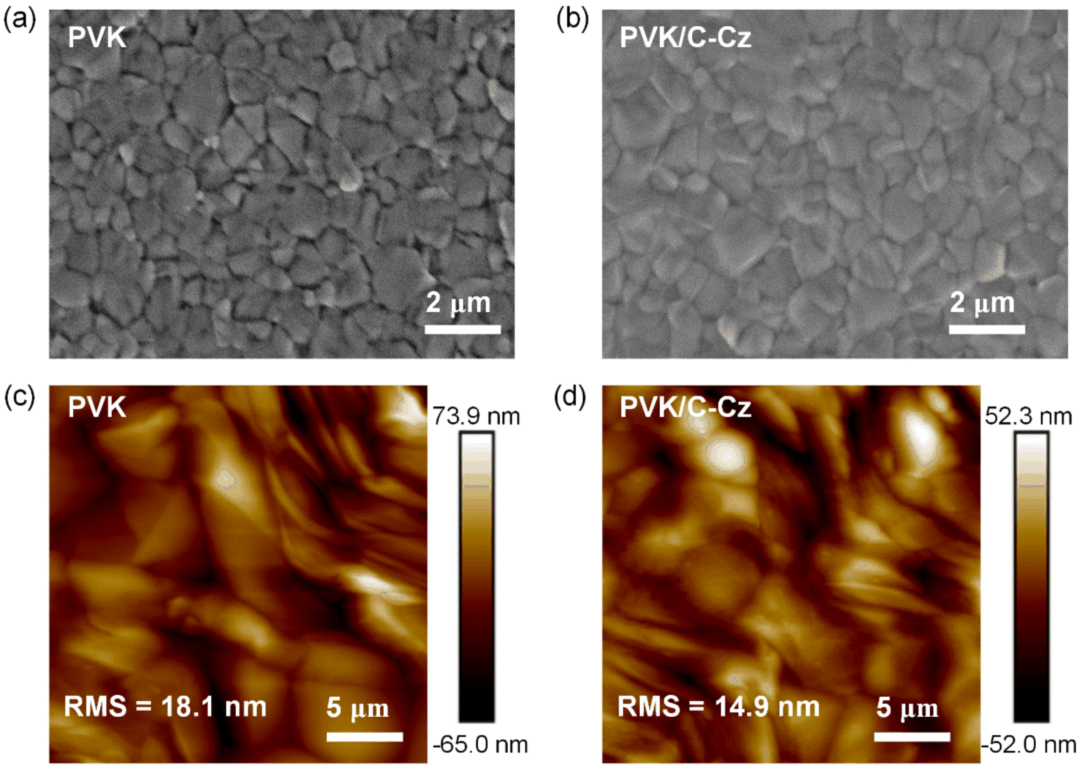

Figure S11. The top-view SEM images of a) pristine perovskite (PVK) film, and b) PVK/C-Cz film. The AFM images of c) PVK film, and d) PVK/C-Cz film.

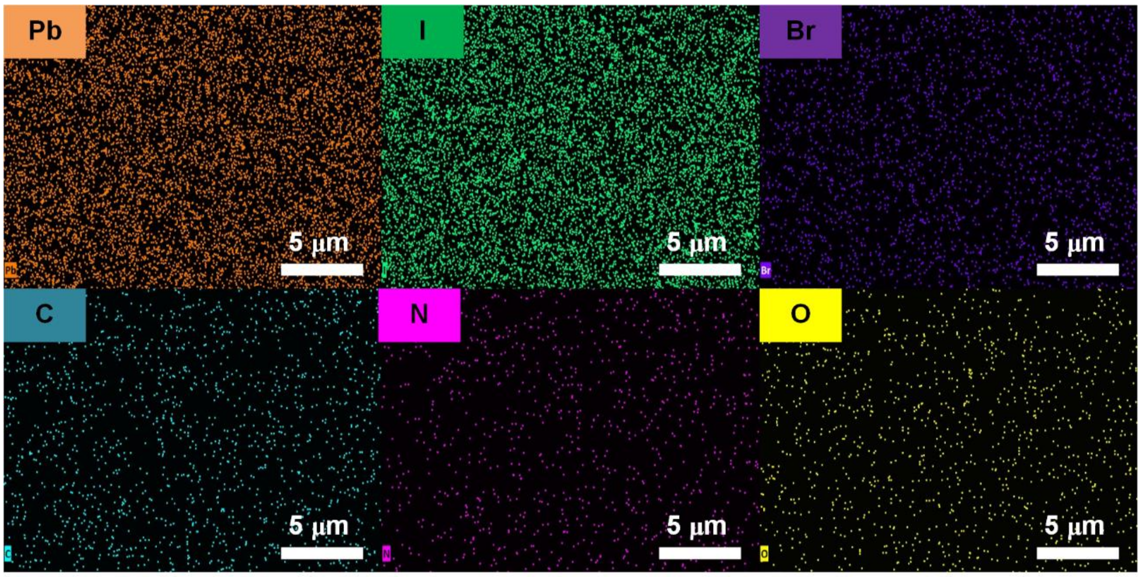

Figure S12. The EDX elemental mappings of C-Cz-modified perovskite film. The images that indicate the atomic symbols correspond to the EDX.

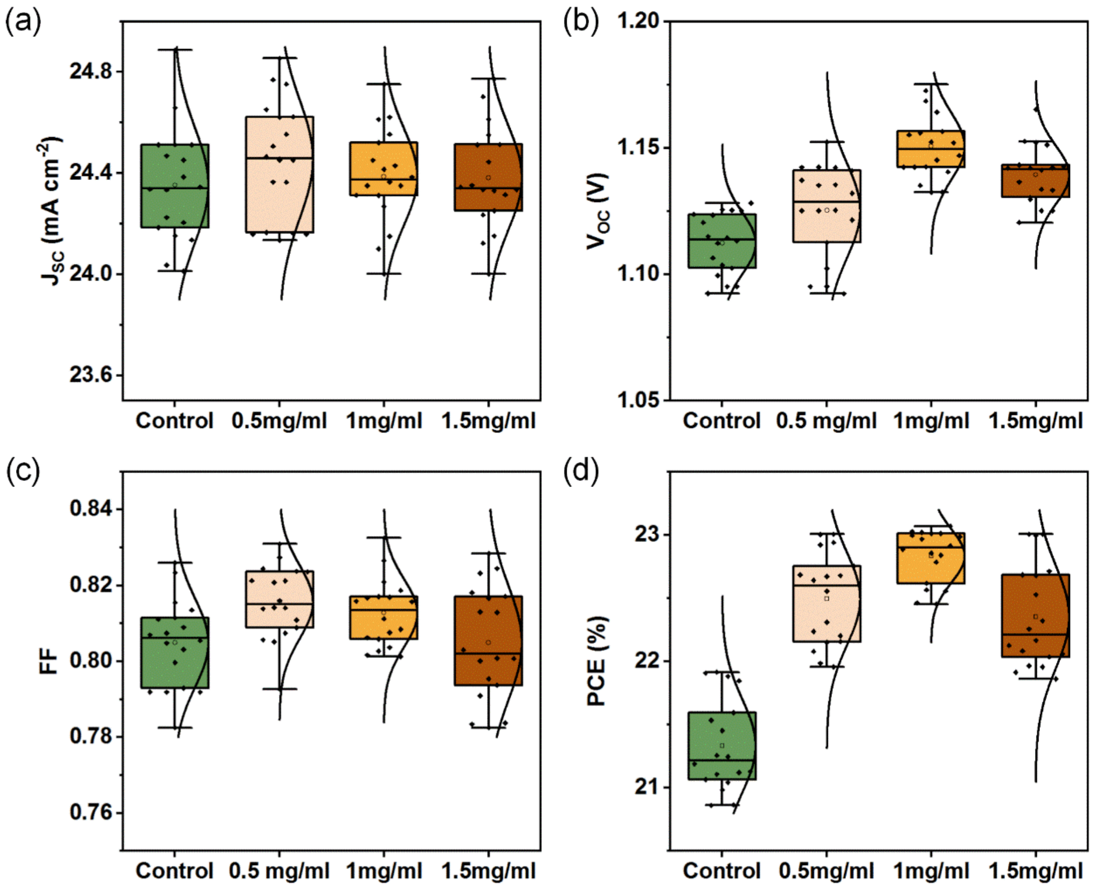

Figure S13. Statistics of the photovoltaic parameters for devices based on **C-Cz** with different concentrations.

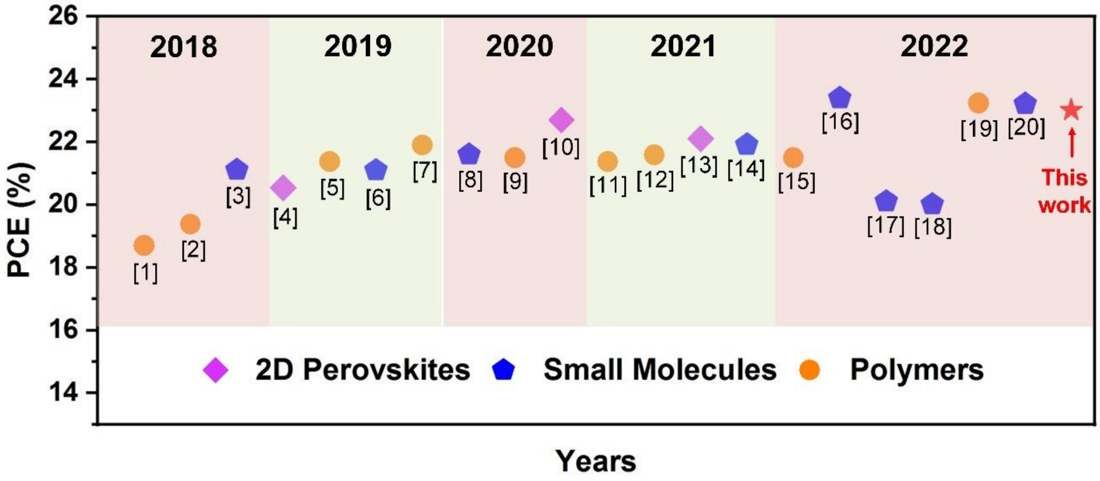

Figure S14. The PCE of PSCs based on one-step method processing with various interfacial engineering strategies in recent years.[5-24]

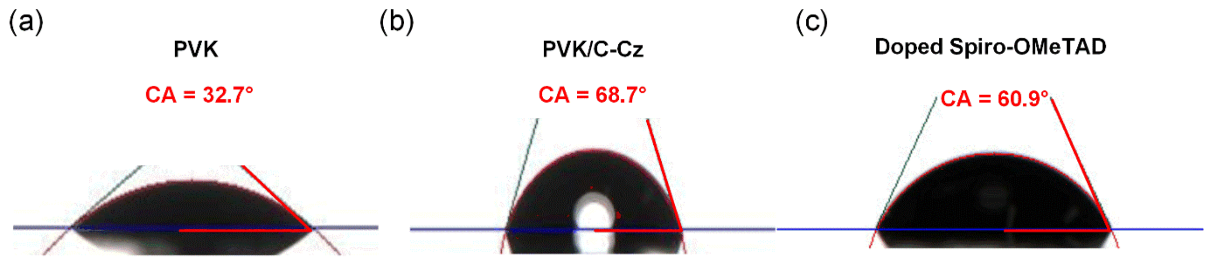

Figure S15. The water contact angles of a) PVK, b) PVK/**C-Cz** and c) doped spiro-OMeTAD films.

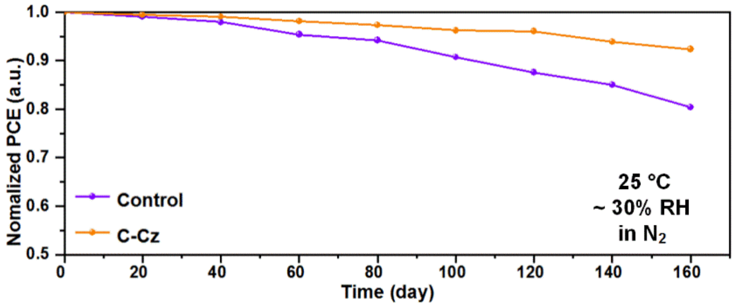

Figure S16. PCE evolution of the unencapsulated PSCs without and with **C-Cz** under N_2 at room temperature (RH = 30 ± 5%).

Table S1. The solubility of C-Cz in different solvents.

Solvents	Solubility of C-Cz (mg/mL)
dichloromethane	95
tetrahydrofuran	102
chloroform	122
toluene	26
chlorobenzene	31
DMF	126
DMSO	131

Table S2. Detailed calculation of cost for the synthesis of 1 g C-Cz.

	Chemicals	Price (US$ g^{-1})	Dosage (g)	Cost (US$)
Step 1	cellulose	0.001	2.5	0.003
	4-methoxytriphenylmethyl chloride	0.26	15.0	3.9
	LiCl	0.17	3.8	0.6
	pyridine	0.04	10	0.4
	DMAc	0.03	100.0	3.0
	methanol	0.008	100.0	0.8
Step 2	DMSO	0.03	150.0	4.5
	NaH	0.07	2.5	0.18
	methyl iodide	0.25	5	1.25
	methanol	0.008	100.0	0.8
Step 3	THF	0.02	100.0	2.0
	concentrated HCl	0.01	2.0	0.02
	acetone	0.02	200.0	4.0
Step 4	carbazole	0.07	2.5	0.18
	1,4-dibromobutane	0.035	5.0	0.18
	Potassium tert-butoxide	0.05	1.8	0.09
	DMF	0.02	10.0	0.2
	THF	0.02	100.0	2.0
	DCM	0.004	300.0	1.2
	petroleum ether	0.004	300.0	1.2
	silica gel	0.0044	200.0	0.88
	NaH	0.07	1.0	0.07
	tetra-*n*-butylammonium iodide	0.1	0.3	0.03
	acetone	0.02	200.0	4.0
	methanol	0.008	200.0	1.6
total cost				33.1

Table S3. Fitting parameters for PL decays and derived time constants.

Samples	τ_1 (ns)	τ_2 (ns)	A_1 (%)	A_2 (%)	τ_{avg} (ns)[a]
Perovskite	31.28	217.68	0.30	0.65	206.09
Perovskite/C-Cz	53.87	362.26	0.26	0.64	344.69

[a] τ_{avg} refers to average lifetime which is calculated as following: $\tau_{avg} = (A_1 \tau_1^2 + A_2 \tau_2^2)/(A_1 \tau_1 + A_2 \tau_2)$.

3. References

[1] P. Taranekar, T. Fulghum, D. Patton, R. Ponnapati, G. Clyde, R. Advincula, *J. Am. Chem. Soc.* **2007**, *129*, 12537.

[2] Frisch, M. J.; Trucks, G. W.; Schlegel, H. B.; Scuseria, G. E.; Robb, M. A.; Cheeseman, J. R.; Scalmani, G.; Barone, V.; Mennucci, B.; Petersson, G. A.et al.Gaussian 09, Revision A.1; Gaussian, Inc.: Wallingford, CT, 2009.

[3] T. Lu, F. Chen, *J. Comput. Chem.* **2012**, *33*, 580.

[4] J. A. Geurst, *Phys. status solidi* **1966**, *15*, 107.

[5] F. Li, J. Yuan, X. Ling, Y. Zhang, Y. Yang, S. H. Cheung, C. H. Y. Ho, X. Gao, W. Ma, *Adv. Funct. Mater.* **2018**, *28*,1706377.

[6] P. L. Qin, G. Yang, Z. W. Ren, S. H. Cheung, S. K. So, L. Chen, J. Hao, J. Hou, G. Li, *Adv. Mater.* **2018**, *30*, 1706126.

[7] Y. Tu, X. Yang, R. Su, D. Luo, Y. Cao, L. Zhao, T. Liu, W. Yang, Y. Zhang, Z. Xu, Q. Liu, J. Wu, Q. Gong, F. Mo, R. Zhu, *Adv. Mater*. **2018**, *30*, 1805085.

[8] Q. Zhou, L. Liang, J. Hu, B. Cao, L. Yang, T. Wu, X. Li, B. Zhang, P. Gao, *Adv. Energy Mater*. **2019**, *9*, 1802595.

[9] C. C. Zhang, Z. K. Wang, S. Yuan, R. Wang, M. Li, M. F. Jimoh, L. S. Liao, Y. Yang, *Adv. Mater.* **2019**, *31*, 1902222.

[10] S. Yang, S. Chen, E. Mosconi, Y. Fang, X. Xiao, C. Wang, Y. Zhou, Z. Yu, J. Zhao, Y. Gao, F. De Angelis, J. Huang, *Science* **2019**, *365*, 473.

[11] F. Tan, H. Tan, M. I. Saidaminov, M. Wei, M. Liu, A. Mei, P. Li, B. Zhang, C. S. Tan, X. Gong, Y. Zhao, A. R. Kirmani, Z. Huang, J. Z. Fan, R. Quintero-Bermudez, J. Kim, Y. Zhao, O. Voznyy, Y. Gao, F. Zhang, L. J. Richter, Z. H. Lu, W. Zhang, E. H. Sargent, *Adv. Mater*. **2019**, *31*, 1807435.

[12] H. Li, J. Shi, J. Deng, Z. Chen, Y. Li, W. Zhao, J. Wu, H. Wu, Y. Luo, D. Li, Q. Meng, *Adv. Mater.* **2020**, *32*, 1907396.

[13] B. Yu, L. Zhang, J. Wu, K. Liu, H. Wu, J. Shi, Y. Luo, D. Li, Z. Bo, Q. Meng, *J. Mater. Chem. A* **2020**, *8*, 1417.

[14] Y. Liu, S. Akin, A. Hinderhofer, F. T. Eickemeyer, H. Zhu, J. Y. Seo, J. Zhang, F. Schreiber, H. Zhang, S. M. Zakeeruddin, A. Hagfeldt, M. I. Dar, M. Grätzel, *Angew. Chemie - Int. Ed*. **2020**, *59*, 15688.

[15] E. Akman, S. Akin, *Adv. Mater*. **2021**, *33*, 2006087.

[16] M. Lyu, S. Park, H. Lee, B. S. Ma, S. H. Park, K. H. Hong, H. Kim, T. S. Kim, J. H. Noh, H. J. Son, N. G. Park, *ACS Appl. Mater. Interfaces* **2021**, *13*, 35595.

[17] K. Ma, H. R. Atapattu, Q. Zhao, Y. Gao, B. P. Finkenauer, K. Wang, K. Chen, S. M. Park, A. H. Coffey, C. Zhu, L. Huang, K. R. Graham, J. Mei, L. Dou, *Adv. Mater.* **2021**, *33*, 2100791.

[18] B. Liu, H. Bi, D. He, L. Bai, W. Wang, H. Yuan, Q. Song, P. Su, Z. Zang, T. Zhou, J. Chen, *ACS Energy Lett.* **2021**, *6*, 2526.

[19] B. Xiao, Y. Qian, X. Li, Y. Tao, Z. Yi, Q. Jiang, Y. Luo, J. Yang, *J. Energy Chem.* **2023**, *76*, 259.

[20] H. Zhang, W. Yu, J. Guo, C. Xu, Z. Ren, K. Liu, G. Yang, M. Qin, J. Huang, Z. Chen, Q. Liang, D. Shen, Z. Wu, Y. Zhang, H. T. Chandran, J. Hao, Y. Zhu, C. sing Lee, X. Lu, Z. Zheng, J. Huang, G. Li, *Adv. Energy Mater.* **2022**, *12*, 2201663.

[21] H. Zhang, Y. Mao, J. Xu, S. Li, F. Guo, L. Zhu, J. Wang, Y. Wu, *J. Mater. Chem. C* **2022**, *10*, 1862.

[22] F. Shini, M. Thambidurai, H. A. Dewi, N. F. Jamaludin, A. Bruno, A. Kanwat, N. Mathews, C. Dang, H. D. Nguyen, *J. Mater. Chem. C* **2022**, *10*, 9044.

[23] B. Zhang, C. Chen, X. Wang, X. Du, D. Liu, X. Sun, Z. Li, L. Hao, C. Gao, Y. Li, Z. Shao, X. Wang, G. Cui, S. Pang, *Angew. Chemie* **2023**, *135*, e202213478.

[24] J. Guo, J. Sun, L. Hu, S. Fang, X. Ling, X. Zhang, Y. Wang, H. Huang, C. Han, C. Cazorla, Y. Yang, D. Chu, T. Wu, J. Yuan, W. Ma, *Adv. Energy Mater.* **2022**, *12*, 2200537.
